# Supplementary material for: Susceptibility of Meropenem-Resistant and/or Carbapenemase-Producing Clinical Isolates of Enterobacterales (Enterobacteriaceae) and Pseudomonas aeruginosa to Ceftazidime-Avibactam and Ceftolozane-Tazobactam as Assessed by In Vitro Testing Methods
Source: Antibiotics (Basel). 2022 Jul 29;11(8):1023. doi: 10.3390/antibiotics11081023 (PMC9405240; doi:10.3390/antibiotics11081023)
Supplement: Supplementary file 1 [file antibiotics-11-01023-s001.zip › antibiotics-1822054-supplementary.pdf]

Table S1. Ceftazidime/avibactam and ceftolozane/tazobactam MIC results of meropenem-resistant and/or carbapenemase-producing clinical isolates.

Isolates by species (*n*)	Susceptibility testing method	Ceftazidime/avibactam (CZA)					Ceftolozane/tazobactam (C/T)				
		MIC value (µg/mL)			MIC category[a]		MIC value (µg/mL)			MIC category[a]	
		Range	MIC_{50}	MIC_{90}	S	R	Range	MIC_{50}	MIC_{90}	S	R
Enterobacteriaceae (153)	BMD	≤1 to >64	4	>64	87	66	4 to >64	>64	>64	0	153
	VITEK 2	≤0.12 to ≥16	4	≥16	85	68	8 to ≥32	≥32	≥32	0	153
	ETEST	0.25 to >256	4	>256	87	66	4 to >256	>256	>256	0	153
Escherichia coli (34)	BMD	≤1 to >64	>64	>64	11	23	4 to >64	>64	>64	0	34
	VITEK 2	≤0.12 to ≥16	≥16	≥16	11	23	8 to ≥32	≥32	≥32	0	34
	ETEST	0.25 to >256	>256	>256	11	23	8 to >256	>256	>256	0	34
Klebsiella pneumoniae (100)	BMD	≤1 to >64	2	>64	75	25	8 to >64	>64	>64	0	100
	VITEK 2	0.25 to ≥16	4	≥16	73	27	16 to ≥32	≥32	≥32	0	100
	ETEST	0.75 to >256	2	>256	75	25	4 to >256	>256	>256	0	100
Other species (19)[b]	BMD	≤1 to >64	>64	>64	1	18	4 to >64	>64	>64	0	19
	VITEK 2	0.25 to ≥16	≥16	≥16	1	18	8 to ≥32	≥32	≥32	0	19
	ETEST	0.5 to >256	>256	>256	1	18	4 to >256	>256	>256	0	19
Pseudomonas aeruginosa (52)	BMD	≤1 to >64	8	>64	26	26	≤0.5 to >64	8	>64	21	31
	VITEK 2	2 to ≥16	8	≥16	26	26	0.5 to ≥32	≥32	≥32	21	31
	ETEST	1 to >256	8	>256	26	26	0.5 to >256	16	>256	21	31
Total (205)	BMD	≤1 to >64	8	>64	113	92	≤0.5 to >64	>64	>64	21	184
	VITEK 2	≤0.12 to ≥16	8	≥16	111	94	0.5 to ≥32	≥32	≥32	21	184
	ETEST	0.25 to >256	4	>256	113	92	0.5 to >256	>256	>256	21	184

MIC, minimum inhibitory concentration; MIC_{50} and MIC_{90}, concentrations required to inhibit 50 and 90% of the isolates, respectively; BMD, broth microdilution.

[a]EUCAST 2021 clinical breakpoints for CZA-susceptible (MIC, ≤8/4 µg/mL) or -resistant (MIC, >8/4 µg/mL) isolates and for CT-susceptible (MIC, ≤2/4 µg/mL for *Enterobacteriaceae*; MIC, ≤4/4 µg/mL for *P. aeruginosa*) or -resistant (MIC, >2/4 µg/mL for *Enterobacteriaceae*; MIC, >4/4 µg/mL for *P. aeruginosa*) isolates were used to interpret isolates' testing results.

[b]Other species include *Citrobacter freundii* (1 isolate), *Enterobacter cloacae* (6 isolates), *Klebsiella aerogenes* (1 isolate), *Klebsiella oxytoca* (9 isolates), *Klebsiella variicola* (1 isolate), and *Raoultella ornithinolytica* (1 isolate).

Table S2. Initial discrepancies between broth microdilution (BMD) and VITEK 2 or ETEST methods and outcome of repeat testing

Organism (isolate designation)	Antimicrobial agent tested	MIC (µg/mL) value with interpretive S/ R category by BMD (reference method)[a]		Initial results[b]			Repeat results[b]			Method as source of error
				MIC (µg/mL)[a]	Interpretive S/R category	Error	MIC (µg/mL)	Interpretive S/R category	Error	
K. pneumoniae (L172)	CZA	≥128	R	8	S	VME	≥16	R	Corrected	VITEK 2
K. pneumoniae (L50)	CZA	64	R	8	S	VME	8	S	VME	VITEK 2
K. pneumoniae (R132)	CZA	4	S	≥16	R	ME	≥16	R	ME	VITEK 2
K. pneumoniae (R219)	CZA	4	S	≥16	R	ME	≥16	R	ME	VITEK 2
K. pneumoniae (R65)	CZA	8	S	≥16	R	ME	≥16	R	ME	VITEK 2
K. pneumoniae (R224)	CZA	8	S	≥16	R	ME	8	S	Corrected	VITEK 2
K. pneumoniae (R235)	CZA	8	S	≥16	R	ME	8	S	Corrected	VITEK 2
P. aeruginosa (R9)	CZA	8	S	≥16	R	ME	8	S	Corrected	VITEK 2
P. aeruginosa (R11)	CZA	4	S	≥16	R	ME	8	S	Corrected	VITEK 2
K. pneumoniae (R229)	CZA	≤1	S	16	R	ME	1	S	Corrected	ETEST
K. pneumoniae (R225)	CZA	2	S	16	R	ME	2	S	Corrected	ETEST
K. pneumoniae (R230)	CZA	4	S	32	R	ME	4	S	Corrected	ETEST
P. aeruginosa (R221)	CZA	2	S	16	R	ME	2	S	Corrected	ETEST
P. aeruginosa (R222)	CZA	4	S	16	R	ME	8	S	Corrected	ETEST
P. aeruginosa (R234)	C/T	4	S	8	R	ME	4	S	Corrected	VITEK 2
P. aeruginosa (R241)	C/T	4	S	16	R	ME	4	S	Corrected	VITEK 2
P. aeruginosa (R220)	C/T	64	R	4	S	VME	32	R	Corrected	ETEST
P. aeruginosa (R221)	C/T	≤0.5	S	1	R	ME	8	S	Corrected	ETEST
P. aeruginosa (R222)	C/T	1	S	8	R	ME	1	S	Corrected	ETEST

CZA, ceftazidime/avibactam; C/T, ceftolozane/tazobactam; MIC, minimum inhibitory concentration; S, susceptible; R, resistant; VME, very major error; ME, major error.

[a]EUCAST (v. 11.0, 2021) clinical breakpoints for *Enterobacteriaceae* (e.g., *Klebsiella pneumoniae*) or *Pseudomonas aeruginosa* were used to interpret MIC values to CZA and C/T, respectively. For convenience, only MIC values of ceftazidime for CZA and of ceftolozane for C/T are shown. It should be recalled that the concentration of avibactam (in CZA) or tazobactam (in C/T) is fixed (i.e., 4 µg/mL).

[b]Repeat testing allowed checking initial discrepant testing results for listed isolates (10 *K. pneumoniae* and 9 *P. aeruginosa*) with Vitek 2 (AST-N397) or Etest methods. Initially, there were 12 MEs (7 with VITEK 2 and 5 with ETEST) and 2 VMEs (both with VITEK 2) regarding CZA. There also were four MEs (2 with VITEK 2 and 2 with ETEST) and one VME (with ETEST) regarding C/T. Repeat testing performed on the isolates resulted in three MEs and one VME, which regarded CZA when tested with Vitek 2 against *K. pneumoniae* isolates.
